# Influence of esophageal temperature probe tip placement on core temperature measurement accuracy in cold environments: a randomized crossover trial with implications for cardiac arrest management

**DOI:** 10.1016/j.resplu.2025.101087

**Published:** 2025-09-04

**Authors:** Giulia Roveri, Tomas Dal Cappello, Alex Hofer, Franziska Breidt, Othmar Kofler, Erik Popp, Hermann Brugger, Simon Rauch

**Affiliations:** a Institute of Mountain Emergency Medicine, Eurac Research, Bolzano, Italy; b Department of Anesthesia and Intensive Care Medicine, Merano Hospital, Italy; c Aiut Alpin Dolomites HEMS, Pontives, Italy; d Heidelberg University, Medical Faculty Heidelberg, Department of Anaesthesiology, Heidelberg, Germany

**Keywords:** Accidental hypothermia, Cold injury, Staging, Triage

## Abstract

**Introduction:**

Accurate core temperature (CT) measurement is critical for staging and management in accidental hypothermia, particularly in cardiac arrest, where it guides extracorporeal rewarming decisions. Esophageal temperature monitoring is considered the reference method in the prehospital setting in patients with a secured airway, provided the probe tip is positioned in the distal third of the esophagus behind the heart. However, the effect of proximal misplacement on measurement accuracy remains unknown. We hypothesized that a probe tip positioned behind the trachea would yield falsely low readings during cold air exposure.

**Methods:**

In this randomized crossover study (May 2024 at Eurac Research, Bolzano, Italy), healthy volunteers underwent nasal esophageal probe placement using a height-based formula. Two probe positions were defined via posteroanterior chest radiographs: correct (behind the heart) and incorrect (5 cm above the tracheal bifurcation). Participants were exposed to –20 °C for 20 min in a climate chamber, once with the probe in the correct and once in the incorrect position, in randomized order, separated by a washout period.

**Results:**

Fifteen participants (7 male, 8 female) completed the study. Mean correct insertion depth was 41.1 (2.5) cm for males and 39.3 (1.5) cm for females. At baseline and throughout –20 °C exposure, mean CT was on average 0.6 °C lower when the probe was incorrectly positioned. Temperature fluctuations were also greater with proximal misplacement.

**Conclusion:**

Proximal misplacement of esophageal probes during cold air exposure results in falsely low and more variable CT readings. This may critically affect triage and treatment, particularly in hypothermic cardiac arrest.

## Introduction

Accidental hypothermia, defined as a core temperature (CT) below 35 °C, is associated with significant morbidity and mortality and can occur year-round, both outdoors and indoors.^1^, ^2^, ^3^, ^4^ Clinical staging using the Revised Swiss System of Hypothermia^5^ is possible based on vital signs, but these often correlate poorly with actual CT,^6^ especially in the presence of confounders such as traumatic brain injury, metabolic disorders, or intoxication. Accurate CT measurement is essential for appropriate staging and to guide transport and management decisions. This requires the use of properly calibrated, low-reading thermometers.^7^

In patients with a secured airway, esophageal temperature monitoring is considered the gold standard.^8^, ^9^ For accurate CT assessment, the probe should be positioned in the distal third of the esophagus, posterior to the heart, where temperature measurements closely correlate with pulmonary artery blood temperature.^10^ However, placing the probe too proximally, such as behind the trachea, may yield inaccurate readings because inhaled cold or warm gases could affect measurements, resulting in falsely low or high temperatures. These inaccuracies may have critical implications for patient triage, particularly in cardiac arrest.^9^ For instance, a falsely low temperature might lead to the erroneous assumption of hypothermic cardiac arrest, prompting prolonged transport under ongoing CPR and initiation of extracorporeal rewarming, which may ultimately be futile.^11^ Conversely, a falsely high temperature could result in missed recognition of hypothermic arrest, thereby denying a potentially life-saving intervention.^12^, ^13^ Despite these implications, the impact of esophageal probe position on the accuracy of CT measurements remains unclear. The primary aim of this study was to determine whether a too proximal placement of the probe tip results in falsely low CT readings during cold air inhalation.

## Material and methods

### Study design and setting

This interventional, randomized, cross-over study was conducted in May 2024 at the terraXcube, an extreme climate simulation centre, at Eurac Research (Bolzano, Italy). The study protocol was approved by the Ethics Institutional Review Board for Clinical Studies of Bolzano (protocol number 27-2024) and prospectively registered at ClinicalTrials.gov (NCT06370676) before recruitment of the first participant. All volunteers gave informed consent to participate in the study. The study was conducted in accordance with Good Clinical Practice and followed the Declaration of Helsinki Guidelines.^14^ The study is reported according to the CONSORT 2010 extension to crossover trials.^15^, ^16^

### Participants

The participants were adults with an American Society of Anesthesiologists (ASA) score ≤ 2.^17^ The exclusion criteria were ASA >3, age <18 or >75 years, pregnancy, signs or symptoms of an acute illness on the day of the study, history of esophageal or nasopharyngeal disorders or allergies to local anesthetics.

### Exposure and measurements

Before the start of the study, each participant underwent a medical history review and physical examination, including weight and height measurements. A 9 French esophageal temperature probe (DeRoyal Industries Inc., Powell, TN, USA) was inserted nasally under local anesthesia for continuous core temperature monitoring. The initial insertion depth was calculated using the formula proposed by Mekjavić et al.^18^:

Insertiondepth(cm)=0.228×standingheight(cm)-0.194.

**Fig. 1:**
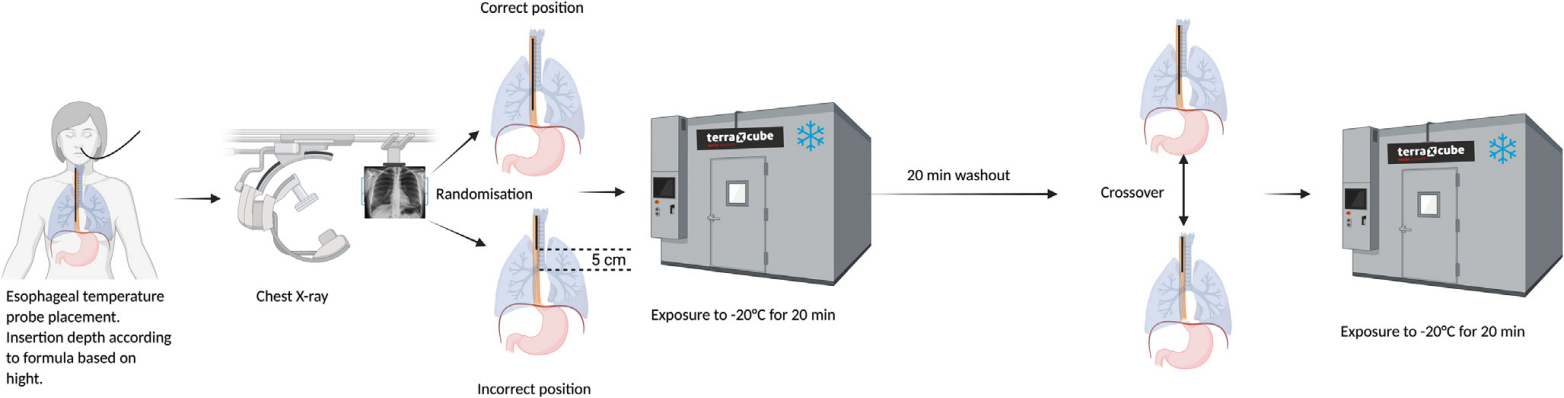
Different steps of the study protocol (created with BioRender.com).

No difficulties or complications occurred during probe placement. Following insertion, a posteroanterior (PA) chest X-ray was performed during inspiration to verify the exact position of the probe tip. Based on the X-ray findings, two positions were defined: correct and incorrect. Correct placement was defined as the tip located behind the right atrium, centrally within the cardiac silhouette, whereas incorrect placement was defined as the tip positioned 5 cm above the tracheal bifurcation (i.e., too proximal, behind the trachea). Based on these findings, the necessary adjustments (i.e., withdrawal or deeper insertion) from the initial position (calculated using the formula) were determined to achieve the predefined correct or incorrect positioning. After a 10-min baseline measurement at ambient room temperature (+20 °C), each participant underwent two 20-minute cold exposure sessions at –20 °C inside the terraXcube while lying supine: one with the probe in the correct position and one in the incorrect position. The allocation sequence was computer-generated and implemented using a concealed randomization list. Investigators responsible for participant enrollment were unaware of upcoming allocations. Participants were blinded to probe position throughout the study. Of the total sample, 7 participants were assigned to the correct–incorrect sequence and 8 participants to the incorrect–correct sequence. A 30-minute washout period at +20 °C was conducted between the two sessions to mitigate potential carryover effects. Esophageal temperature was recorded every 30 s throughout both cold exposure sessions. To prevent hypothermia, participants were provided with warm clothing, insulation, and thermal blankets. The different steps of the study protocol are illustrated in Fig. 1.

### Outcomes

The primary outcome was the difference in temperature readings between correctly positioned and incorrectly positioned esophageal probes. Secondary outcomes included i) comparing fluctuations in the temperature readings between the two positions and ii) assessing placement accuracy by comparing the probe position calculated using the formula by Mekjavić et al.^18^ with the correct insertion depth determined from the X-ray.

### Sample size calculation and statistical methods

A temperature difference of ≥0.5 °C compared to the correctly positioned probe was considered clinically relevant, in line with prior literature demonstrating that such discrepancies can influence clinical decision-making in perioperative and critical care settings.^19^, ^20^, ^21^ To detect such a difference, assuming a standard deviation of 0.5 °C for the within-subject differences (correct vs. incorrect probe position), a sample size of 13 participants was calculated for a Type I error of 0.05 (one-sided) and a statistical power of 95 %.

The statistical analysis was carried out using SPSS software version 29 (IBM Corp., Armonk, NY, USA). Normal distribution was assessed by means of Shapiro-Wilk test and normal Q-Q plots. Paired *t*-test was used to compare the temperature at the correct placement of the probe with the temperature at the incorrect placement at defined timepoints, adjusting p-values by means of Benjamini-Hochberg method. Data were reported as mean (standard deviation, SD) and *p* < 0.05 (one-sided) was considered as statistically significant.

## Results

Fifteen participants (7 male, 8 female) were enrolled, and all completed the study without dropouts. Demographic and baseline characteristics are presented in Table 1.

**Table 1:** Demographic data and baseline characteristics of the participants. Data are presented as mean (standard deviation). BMI − body mass index.

**Parameter**	**Male (N = 7)**	**Female (N = 8)**
Age, years	36 (12)	31 (10)
Weight, kg	83 (14)	66 (6)
Height, cm	178 (5)	170 (4)
BMI, kg/m^2^	26.0 (3.8)	23.1 (2.4)

### Comparison of core temperature measurements between correct and incorrect esophageal probe positioning

**Fig. 2:**
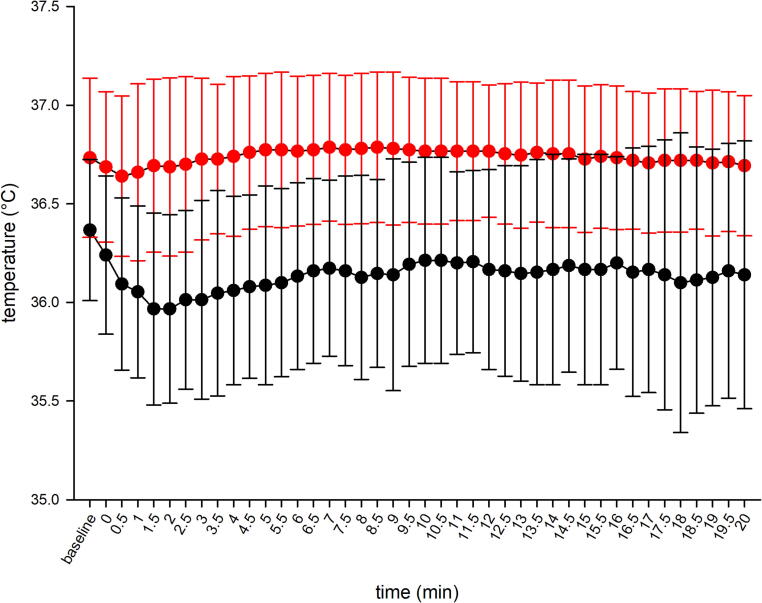
Mean of measured temperature at baseline (+20 °C) and during cold exposure (−20 °C). Error bars represent standard deviation, red colour indicates the correct position and black colour indicates the incorrect esophageal probe position. (For interpretation of the references to colour in this figure legend, the reader is referred to the web version of this article.)

At baseline and at all timepoints during exposure to −20 °C, mean CT was significantly lower when the probe was incorrectly positioned (Table 2, Fig. 2). On average, measurements from the incorrect position were 0.6 °C lower than those obtained from the correctly positioned probe.

**Table 2:** Differences in temperature between correct and incorrect esophageal probe position at defined timepoints (at baseline (+20 °C) and during cold exposure (−20 °C)). Values are reported as mean (standard deviation) and *p*-values are adjusted with Benjamini-Hochberg method.

Timepoint	Temperature correct placement	Temperature incorrect placement	Difference	*p*-value
Baseline	36.7 (0.4) °C	36.4 (0.4) °C	0.4 (0.3) °C	<0.001
0 min	36.7 (0.4) °C	36.2 (0.4) °C	0.4 (0.4) °C	<0.001
5 min	36.8 (0.4) °C	36.1 (0.5) °C	0.7 (0.4) °C	<0.001
10 min	36.8 (0.4) °C	36.2 (0.5) °C	0.6 (0.4) °C	<0.001
15 min	36.7 (0.4) °C	36.2 (0.6) °C	0.6 (0.5) °C	<0.001
20 min	36.7 (0.4) °C	36.1 (0.7) °C	0.6 (0.6) °C	0.001

### Temperature reading fluctuations

**Fig. 3:**
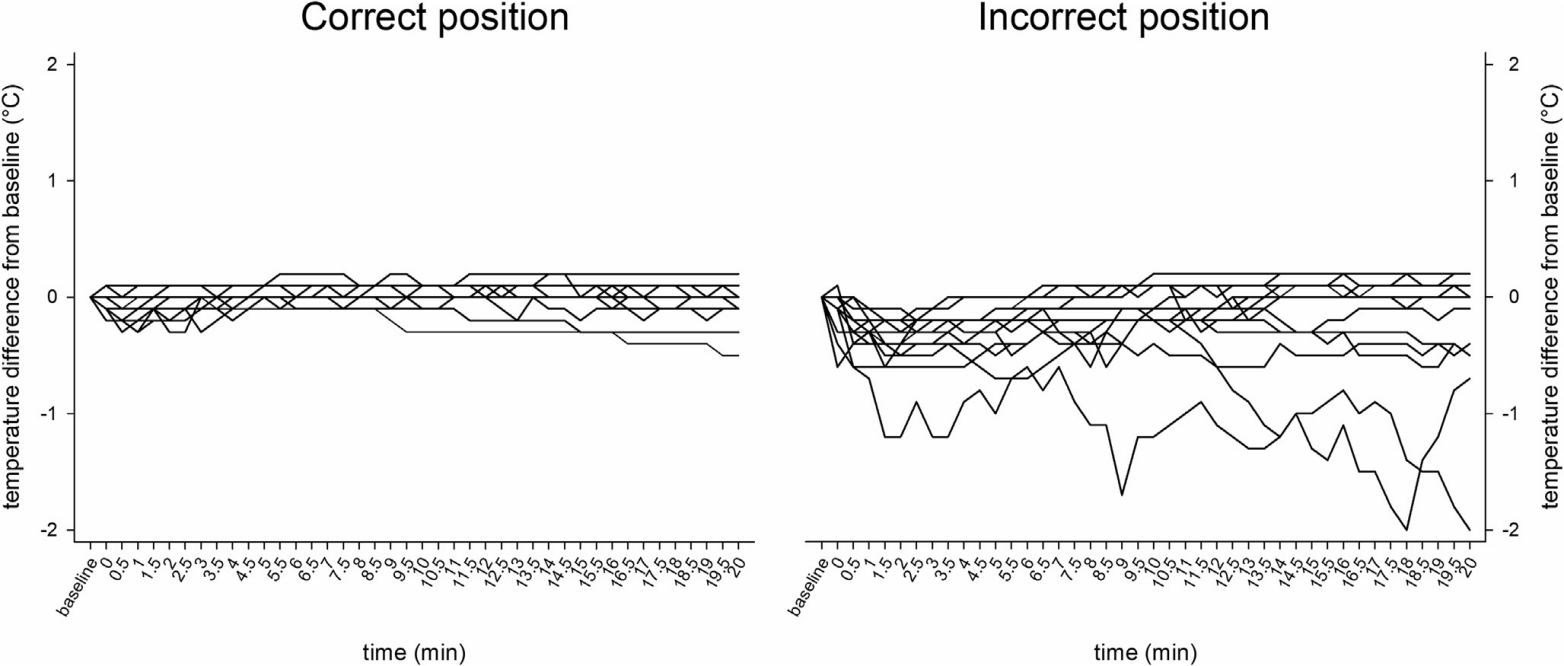
Temperature difference at baseline and during cold exposure (−20 °C) for each participant with correct vs. incorrect esophageal probe placement.

Fluctuations from baseline temperature during cold exposure for each participant are shown in Fig. 3. Variability was greater with the probe in the incorrect position, ranging at 5 min from –0.1 °C to 0.1 °C when correctly placed versus –1.0 °C to 0 °C when incorrect, and at 10 min from –0.3 °C to 0.1 °C versus –1.2 °C to 0.2 °C, respectively.

### Insertion depths and esophageal probe placement accuracy

**Fig. 4:**
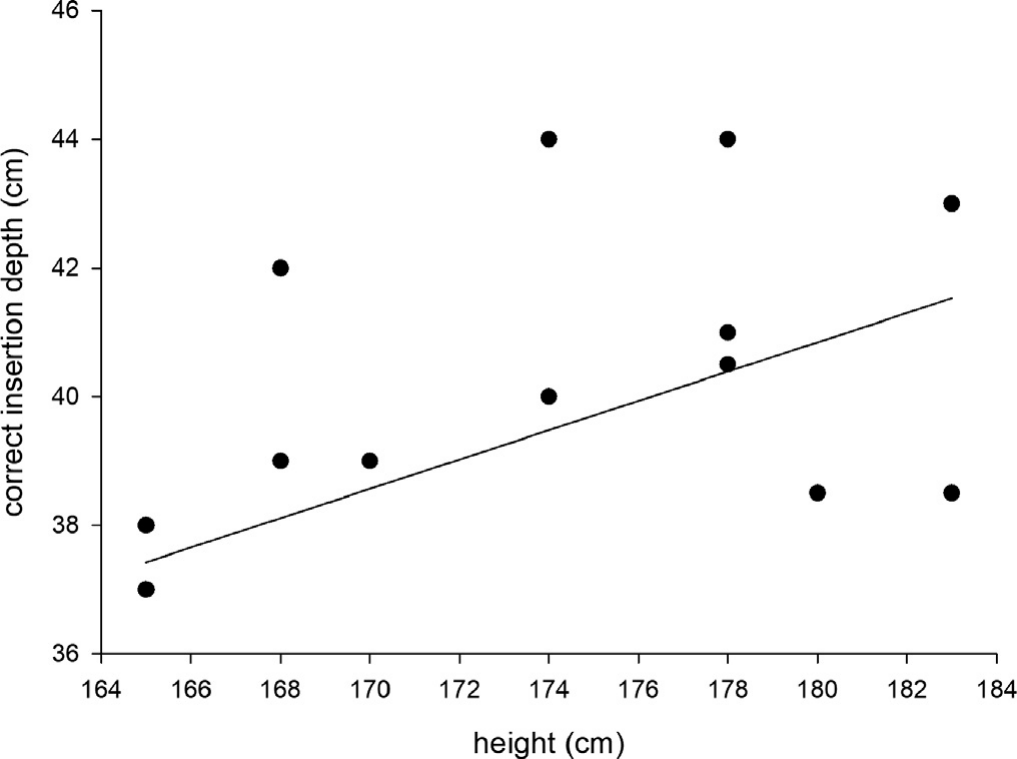
Scatterplot of height and correct insertion depth. The straight line represents the insertion depth calculated using the formula by Mekjavić et al.^18^

Using the insertion depth formula by Mekjavić et al.,^18^ radiographically confirmed correct positioning of the esophageal temperature probe (within a tolerance of ±1 cm) was achieved in 60 % of participants. Deviations from the optimal position ranged from –3.0 cm to +4.5 cm (Fig. 4).

#### Male participants

The mean correct insertion depth was 41.1 (2.5) cm (range: 38.5–44.0  cm). The minimal insertion depth, as the distance required to pass the tracheal bifurcation, ranged from 31.0 to 35.0 cm (mean 33.8 cm; SD 1.55 cm). The maximum insertion depth, defined as the distance to the gastroesophageal junction, ranged from 44.0 to 51.0  cm (mean 47.9; SD 2.9  cm).

#### Female participants

The mean correct insertion depth was 39.3 (1.5) cm (range: 37–42  cm). The minimal insertion depth ranged from 28.0 to 33.0 cm (mean 31.1 cm; SD 1.56 cm), while the maximal insertion depth ranged from 42.0 to 49.0 cm (mean 45.7 cm; SD 2.3 cm).

## Discussion

In this study, we found that esophageal temperature probes positioned too proximally (i.e., behind the trachea) lead to falsely low core temperature (CT) readings and greater measurement fluctuations during ambient and, more considerably, during cold air inhalation.

Accurate CT measurement in hypothermic patients is critical for both triage and treatment decisions, especially in cardiac arrest (CA) cases. Hypothermia is considered a reversible cause of CA^9^ and, when treated with extracorporeal rewarming, offers significantly higher survival rates with favorable neurological outcomes compared to non-hypothermic CA.^1^, ^8^, ^12^, ^13^ However, differentiating between hypothermic and non-hypothermic CA can be challenging, especially in prehospital settings, and CT can serve as an important discriminative tool. In otherwise healthy individuals, hypothermia rarely causes cardiac arrest unless CT falls below 30 °C, whereas in older, multimorbid patients, hypothermia may contribute to cardiac arrest at temperatures below 32 °C.^9^, ^11^ Reliable temperature measurement is key not only for diagnosis but also for prognostication. For instance, the HOPE score, the recommended tool for predicting survival in hypothermic CA patients undergoing extracorporeal life support rewarming,^9^, ^22^, ^23^, ^24^ relies heavily on precise CT readings. Even small variations can influence clinical decisions: for example, a 60-year-old male with a non-asphyctic CA, 45 min of CPR, and a potassium level of 5.0  mmol/L would have a HOPE survival probability of 11 % at 29.5 °C, but only 9 % at 30.0 °C. Given that the current decision threshold for extracorporeal rewarming is 10 %,^25^ a difference of just 0.5 °C could decisively affect management. This illustrates the critical importance of accurate CT measurement.

Esophageal temperature measurement is considered the gold standard for patients with a secured airway in prehospital care.^7^, ^10^ To reflect central blood temperature, the probe should be placed in the lower third of the esophagus, typically behind the heart.^10^ Given the clinical importance of accurate CT measurement, this study tested the hypothesis that too proximal placement of the esophageal probe tip results in inaccurate CT readings, particularly during cold air exposure, as it often occurs when ventilating patients in cold environments in prehospital settings. We observed that when the probe was incorrectly placed behind the trachea, the recorded temperature was, on average, 0.6 °C lower than with correct placement. Additionally, temperature fluctuations were greater in the incorrectly placed group. This supports our hypothesis that cold air in the trachea affects temperature readings in the proximal esophagus due to their close anatomical proximity. While a mean difference of 0.6 °C may appear minor, the combination of this underestimation and increased variability can meaningfully impact decision-making, as demonstrated by the HOPE score example. These findings emphasize the importance of precise probe placement for reliable CT readings.

Several non-radiographic methods for assessing esophageal probe placement have been proposed, typically calculating insertion depth based on patient height. However, formulas such as that by Mekjavić et al.^18^ are often impractical in emergency or prehospital settings, where height is usually only roughly estimated rather than precisely measured. To establish a pragmatic and safe insertion depth for esophageal temperature probes in these settings, we defined the target position as at least 4 cm distal to the tracheal bifurcation and at least 4 cm proximal to the gastroesophageal junction. Based on our radiographic measurements, we found that using a single insertion depth of 38 cm for patients ≤170 cm in height and 40 cm for patients >170 cm in height ensures consistent placement within this safe window. For oral insertion, as commonly performed in emergency settings, the insertion depth should be reduced by approximately 5 cm compared to nasal insertion.^26^ Using a landmark technique could represent an alternative to height-based insertion depths to account for interindividual anatomical variations; however, no validated landmark technique for esophageal probe placement has been evaluated to date.

### Limitations

A key limitation of this study is that it was conducted in a relatively small sample size of healthy, normothermic participants with spontaneous breathing, which may limit direct generalization to hypothermic patients, particularly those who are intubated or passively ventilated. However, the underlying physiological mechanisms remain relevant and, in intubated patients, air delivered through ventilation may amplify tracheal cooling, making proximal esophageal placement even less reliable. Therefore, our findings could even underestimate the degree of error introduced by incorrect probe placement in actual hypothermic scenarios. Additionally, the proposed fixed insertion depths were derived from a small, healthy volunteer sample and may not fully account for anatomical variations in patients outside the height range of our participants or in cases of obesity.

## Conclusion

Incorrect placement of esophageal temperature probes, particularly when positioned too proximally behind the trachea, results in falsely low and more variable CT readings during cold air exposure. Such inaccuracies can significantly influence triage, prognostication, and treatment decisions, especially in hypothermic cardiac arrest patients, where precise CT is critical to guiding extracorporeal rewarming interventions.

## Access to data and data analysis

Giulia Roveri, Simon Rauch and Tomas Dal Cappello had full access to all the data in the study and take responsibility for the integrity of the data and the accuracy of the data analysis.

## Availability of data

All relevant data are included within the article.

## Declaration of Generative AI and AI-assisted technologies in the writing process

During the preparation of this work the author(s) used ChatGPT for language optimisation. After using this tool/service, the author(s) reviewed and edited the content as needed and take(s) full responsibility for the content of the publication.

## CRediT authorship contribution statement

**Giulia Roveri:** Writing – review & editing, Writing – original draft, Project administration, Methodology, Investigation, Formal analysis, Data curation, Conceptualization. **Tomas Dal Cappello:** Writing – review & editing, Formal analysis, Data curation. **Alex Hofer:** Writing – review & editing, Methodology, Investigation, Data curation, Conceptualization. **Franziska Breidt:** Writing – review & editing, Methodology, Investigation, Data curation, Conceptualization. **Othmar Kofler:** Writing – review & editing, Validation. **Erik Popp:** Writing – review & editing, Methodology. **Hermann Brugger:** Writing – review & editing, Supervision, Methodology, Conceptualization. **Simon Rauch:** Writing – review & editing, Writing – original draft, Validation, Supervision, Project administration, Methodology, Formal analysis, Data curation, Conceptualization.

## Funding

No funding was received for this study.

## Declaration of competing interest

The authors declare that they have no known competing financial interests or personal relationships that could have appeared to influence the work reported in this paper.
